# Stacked Model-Based Classification of Parkinson’s Disease Patients Using Imaging Biomarker Data

**DOI:** 10.3390/bios12080579

**Published:** 2022-07-29

**Authors:** Jigna Hathaliya, Hetav Modi, Rajesh Gupta, Sudeep Tanwar, Fayez Alqahtani, Magdy Elghatwary, Bogdan-Constantin Neagu, Maria Simona Raboaca

**Affiliations:** 1 Department of Computer Science and Engineering, Institute of Technology, Nirma University, Ahmedabad 382481, India; 19ftphde36@nirmauni.ac.in (J.H.); 19bce078@nirmuni.ac.in (H.M.); 18ftvphde31@nirmauni.ac.in (R.G.); 2 Software Engineering Department, College of Computer and Information Sciences, King Saud University, Riyadh 12372, Saudi Arabia; fhalqahtani@ksu.edu.sa; 3 Biomedical Technology Department, College of Applied Medical Sciences, King Saud University, Riyadh 12372, Saudi Arabia; melghatwary@ksu.edu.sa; 4 Power Engineering Department, Gheorghe Asachi Technical University of Iasi, 700050 Iasi, Romania; bogdan.neagu@tuiasi.ro; 5 National Research and Development Institute for Cryogenic and Isotopic Technologies—ICSI Rm. Valcea, Uz-inei Street, No. 4, P.O. Box 7 Raureni, 240050 Râmnicu Vâlcea, Romania

**Keywords:** Parkinson’s disease, imaging biomarkers, machine learning, stacked model, classification, dopamine level, disease progression

## Abstract

Parkinson’s disease (PSD) is a neurological disorder of the brain where nigrostriatal integrity functions lead to motor and non-motor-based symptoms. Doctors can assess the patient based on the patient’s history and symptoms; however, the symptoms are similar in various neurodegenerative diseases, such as progressive supranuclear palsy (PSP), multiple system atrophy—parkinsonian type (MSA), essential tremor, and Parkinson’s tremor. Thus, sometimes it is difficult to identify a patient’s disease based on his or her symptoms. To address the issue, we have used neuroimaging biomarkers to analyze dopamine deficiency in the brains of subjects. We generated the different patterns of dopamine levels inside the brain, which identified the severity of the disease and helped us to measure the disease progression of the patients. For the classification of the subjects, we used machine learning (ML) algorithms for a multivariate classification of the subjects using neuroimaging biomarkers data. In this paper, we propose a stacked machine learning (ML)-based classification model to identify the HC and PSD subjects. In this stacked model, meta learners can learn and combine the predictions from various ML algorithms, such as K-nearest neighbor (KNN), random forest algorithm (RFA), and Gaussian naive Bayes (GANB) to achieve a high performance model. The proposed model showed 92.5% accuracy, outperforming traditional schemes.

## 1. Introduction

Parkinson’s disease (PSD) is a progressive neurodegenerative disease of the brain which causes the loss of dopamine neurons at every stage of the disease. It manifests motor and non-motor symptoms, such as depression; anxiety; insomnia; movement-based disorders such as bradykinesia; and speech, rigidity, and tremor issues [1]. The PSD symptoms are similar to other disorders, such as corticobasal syndrome and essential tremor. Thus, it becomes challenging to identify the PSD patients from other disorders and HC [2]. The diagnosis of a disease is usually evaluated using clinical and laboratory tests, so there is a chance of misdiagnosis. Moreover, the clinical tests are not giving fruitful results to identify the disease’s progression at every stage. To address this issue, we have used imaging biomarkers to accurately diagnose the patient [3,4].

Imaging biomarkers are used to identify the various disease patterns and help to measure the level of dopamine, glucose metabolism, dopamine level degeneration, etc. [5]. The various imaging biomarkers are: (i) structural magnetic resonance imaging (MRI) is used to analyze the susceptibility of weighted sequences for the voxel and volumetric-based morphometric, (ii) diffusion tensor MRI is used to evaluate microstructural integrity and white matter tract injury, (iii) proton magnetic resonance spectroscopy (PMRS) is used to quantify the proton levels of brain metabolites, (iv) single-photon emission computed tomography (SPECT) is used to identify the disease patterns of CSF and dopamine level degeneration level inside the brain, and (v) positron emission tomography (PET) is used to recognize the glucose metabolism, neuroinflammation, nigrostriatal integrity functions, and molecular imaging for amyloid and tau [6,7]. We used imaging biomarkers to make an early and accurate diagnosis decision and analyze a particular patient’s disease progression at every single visit.

Imaging biomarkers are imaging modalities that visualize the different patterns inside the brain. Due to these patterns, doctors can easily know the spread area of disease. We have collected the medical images in terms of biomarkers from the PPMI dataset. We have used an ML-based stacked model to extract the biological features from the image and visualize patterns inside the brain. This aids in knowing the dopamine level inside the brain. The deficiency of dopamine levels identifies the PSD and HC patients from the dataset. These patterns also detected the spread area to know the progression of the disease inside the brain. The disease progression level (DPL) of each patient is measured at every subsequent hospital visit and analyzes the critical level of the patient [8]. It is helpful to predict the risk level of a patient. Based on risk, a doctor can suggest the treatment and change the medicine accordingly. In addition, the disease progression is helpful for a patient to take some preventive measures regarding the disease [9].

Presently, the diagnosis is based on the patient’s history and symptoms. Doctors can manually assess the patient and generate a diagnosis report. However, the various diseases may have similar symptoms in the medical diagnosis, so clinical diagnosis may not yield accurate results. Neuroimaging biomarkers recommend the incorporation of biomarkers for PSD diagnosis. The curve region is identified from a group in the individual level classification of a patient, and multivariate classification can employ machine learning (ML) techniques for imaging biomarkers. A combination of the ML algorithms is being designed to extract useful information for classification and accurate diagnosis [10]. Thus, we have designed the stacked an ML model, which can help neuroimaging technologies identify disease patterns and provide better healthcare and treatment to patients in a timely manner. Thus, early detection of disease helps their patients take some preventive measures that reduce their risk levels. Based on the patient’s symptoms and disease patterns, the ML algorithm can identify the dopamine level inside the brain and accurately classify the healthy control (HC) and PSD patients from the imaging biomarkers.

In this paper, we designed a stacked ML model in which a meta learner was used to combine the predictions of various ML algorithms [11], such as K nearest neighbor (KNN) algorithm, random forest algorithm (RFA), and Gaussian naive Bayes (GANB), and improve the model’s performance. It is useful for the accurate diagnosis of patients. For model training, the images of patients were collected using the PPMI image dataset, which have consistent image size, imaging modality, and 3-dimensional (3D) scanned equipment settings. After data collection, data were split into the training and testing datasets. Data pre-processing is an essential requirement to normalize and scale the data before feeding that data into a stacked model. First, we trained our model using the KNN and compared our results with each trained model. Afterward, we used a meta learner which combines the predictions from KNN, RFA, and GANB ML algorithms. Finally, we selected a logistic regression algorithm as a meta learner. This algorithm combines the predictions from all three ML algorithms and provides a significant model output.

### 1.1. Motivation

In state-of-the-art research works, the doctors could assess and generate a clinical diagnosis report based on the symptoms and history of that patient. Due to similar symptoms of various diseases, such as PSD, PSP, and MSA—essential tremor and Parkinson tremor—the possibility of misdiagnosis is there. To address this issue, imaging biomarkers are used to visualize the patterns inside the brain. To improve the diagnosis and to know the disease progression of the patient, many authors [12,13,14,15,16] have used ML models to extract the biological features from medical images and classify them. They have used various AI and ML algorithms, such as support vector machine (SVM) and decision tree algorithm, to classify the PSD, PSP, MSA, and HC subjects, but did not measure the patients’ disease progression. Motivated by this, we proposed an ML stacked classification model in which meta learners can learn from several ML algorithms and combine its predictions to enhance the performance of the proposed model. We also measured the disease progression at each subsequent hospital visit which helps to identify the risk level and provide better delivery care to the patient.

### 1.2. Contributions

The research contributions of this paper are as follows.

- We have used neuroimaging biomarkers to extract the deficiency level of dopamine inside the brain and measure the disease progression at every subsequent visit to the hospital.
- We proposed a stacked ML-based classification model to identify the HC and PSD subjects from the dataset.
- We evaluated the performance of the proposed stacked model using various evaluation metrics, such as accuracy, precision, specificity, and sensitivity.

### 1.3. Organization

The rest of the paper is organized as follows. Section 2 describes the related work. The system and problem formulation are presented in Section 3. Section 4 presents the proposed ML stacked model-based PSD classification. Section 5 defines the performance evaluation. At the end, paper is summarized in Section 6.

## 2. Related Work

This section presents the current work related to imaging-biomarkers-based feature extraction inside the human brain of the patient. Earlier, imaging-based modalities were used to measure dopamine level, fluid, glucose metabolism, and many more. Various imaging biomarkers, such as fMRI, SMRI, PET, and SPECT, are associated with different roles in extracting ROI features for the correct diagnosis of disease and help us to know about the disease progression of the patient. Many authors have used various imaging biomarkers to extract the features from the image to measure the patient’s disease progression. For example, the authors of [12] presented the retina-based biomarkers to differentiate PSD, HC, and Alzheimer’s patients. They have used SVM and linear regression to classify the patient with assigned labels. Early diagnosis helps patients take preventive measures early to prevent the risk associated with the disease. Their proposed scheme managed to achieve a classification accuracy of 87.7%.

Then, Mangesius et al. [14] used a decision algorithm to distinguish parkinsonism patients from imaging biomarkers. They analyzed the NFL serum and MR planimetric level inside the brain to differentiate the MSA, PSP PSD, and HC patients. They used a decision tree algorithm to train the model and achieved an accuracy of 83.7% from a diagnostic test but did not measure the patient’s disease progression. Later, Kathuria et al. [16] presented the 3T MRI nigrosome images to diagnose PSD subjects. They found a negative association between clinical features and loss of nigrosome inside the brain.This proposed scheme accurately diagnoses degeneration PSD syndromes but does not differentiate between idiopathic and atypical parkinsonism.

Pereira et al. [13] described a PSD classification scheme using imaging biomarkers. They used medical imaging biomarkers such as MRI and SPECT to classify patients from PSD, HC, scans without evidence of dopaminergic deficit (SWEDD), and other similar characteristic-based diseases. This scheme uses a convolutional neural network (CNN) to identify the patterns in regions of interest regarding the PSD from the imaging modalities. Additionally, the classification scheme analyzes the basal ganglia midbrain and differentiates the control and PSD patients, and PSD and SWEDD patients, but cannot differentiate the SWEDD and control patients because SWEDD patients do not have any dopamine deficit. Then, Lin et al. [15] extracted the biological features and measured the disease progression using imaging biomarkers. They analyzed the plasma neurofilament light chain (NFL) with electrochemiluminescence immunoassay levels inside the brain. They also measured the changes in Unified Parkinson’s Disease Rating Scale (UPDRS) and motor score with the MMSE score, which is used to evaluate motor and cognition-based disease progression. In addition, the classification scheme classifies the patient as HC, PSD, or MSA with imaging biomarkers to identify the patient’s disease progression.

To solve the aforementioned issue, we proposed a stacked model-based classification scheme. The proposed scheme classifies the PSD and HC patients using imaging biomarkers. We found that most research has been done using individual ML algorithms for classification. Therefore, we used a stacked model with logistic regression as a meta learner. It can learn from various KNN, RFA, and GANB algorithms and combine the results to achieve an accurate performance. In this paper, we consider dopamine level as our region of interest; we extracted the dopamine level patterns from the biomarkers during the model building. Based on the patterns of biomarkers, the proposed scheme classifies the patients and HC accurately.

Table 1 shows a comparative analysis of the existing imaging biomarker-based schemes and the proposed one considering the parameters such as objectives, the algorithm used, results achieved, pros, and cons.

## 3. System Model and Mathematical Problem Formulation

This section elaborates the system and the mathematical formulation of problem.

### System Model

**Figure 1:**
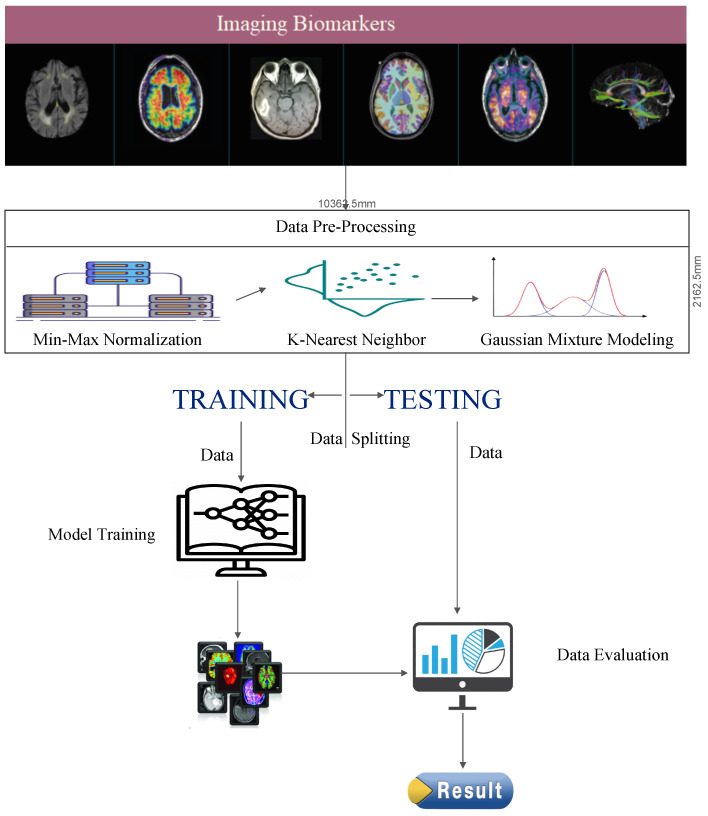
The system.

Figure 1 shows the proposed system. Initially, imaging biomarkers were obtained using the PPMI dataset [17], which contains the information about the image size, imaging modality, and 3D scanning equipment settings. First, we divided the PPMI dataset into training and testing data. Further, in the pre-processing stage, the data were first normalized using min-max normalization, and then, after data reduction was explored using different techniques, such as feature agglomeration (FA), principle component analysis (PCA) and Gaussian mixture modeling (GMM), we chose the best representation of the data from the discussed techniques. After preprocessing, we fed that normalized data into a stacked ML model. The mathematical formulation of the data preprocessing is as follows:

(1) $$X^{\prime }=\frac{X-\min(X)}{\max(X)-\min(X)}$$

Equation (1) shows the formulation for min-max normalization.

We used euclidean distance as the metric to calculate the linkage between the clusters in FA to reduce the dimensionality. The euclidean distance between two points is calculated according to the following equation:

(2) $$d\left(m,n\right)=\sqrt{\sum_{i=0}^{c}{\left(n_{i}-m_{i}\right)}^{2}}$$

where d(m,n) represents the euclidean distance, which is the square root of the sum of squared differences in their elements.

Another method we used for dimensionality reduction was PCA. This transformed the data to a new sample dataset of smaller dimensions. First, we converted data in a matrix form, and then calculated the mean using the following equation:

(3) $$\bar{X}=\frac{1}{N}\sum_{k=1}^{N}X_{k}$$

where $\bar{X}$ denotes the input data, *X_k_* presents the *k*th item of the data, and *N* describes the number of items. We centered the values for each attribute based on the calculated mean.

After the mean calculation, the data values were used to calculate the covariance matrix. Covariance was computed using the following equation:

(4) $$C=\frac{1}{N}\sum_{k=1}^{n}\left(X_{k}-\bar{X}\right){\left(X_{k}-\bar{X}\right)}^{T}$$

where *T* denotes the transpose of the matrix.

GMM was another method used to reduce dimensionality of the data. GMM is calculated using the following equation:

(5) $$G\left(a_{i}|b\right)=\frac{1}{\sqrt{2\pi \sigma_{y}^{2}}}exp(-\frac{{\left(a_{i}-\mu_{y}\right)}^{2}}{2\sigma_{y}^{2}})$$

where μ denotes a dimensional vector of the distribution and σ is the d × d co-variance matrix. We used the minimum reconstruction error to choose among the proposed dimensionality methods.

Initially, we trained the model using KNN, RFA, and GANB classifiers as base learners and then used logistic regression as a meta learner to enhance the performance of our model. It was used to combine the predictions of the KNN, RFA, and GANB ML techniques. KNN prediction probability is computed using the following equation:

(6) $$P\left(b=l|J=j\right)=\frac{1}{k}\sum_{i\in C}I\left(b^{\left(i\right)}=l\right)$$

Equation (6) computes the probability of KNN, and each sample *j* gets assigned a class with the largest probability. We calculated the euclidean distance between all the points and assigned it to the class with the highest number of data points out all the classes of K neighbors. Here, *l* represents the labels and *I* represents the set of points trained for KNN.

RFA prediction was calculated using the following equation:

(7) $$G=1-\sum_{k=1}^{C}{\left(f_{k}\right)}^{2}$$

Equation (7) uses the class and probability to define the Gini index *G* of each branch of node, *C* denotes the number of classes, and $f_{k}$ presents the frequency of the class in the dataset. Gini index is used to calculate the entropy, which is defined as follows.

(8) $$E_{p}=\sum_{k=1}^{C}-\left(f_{k}\right)\times log_{2}\left(f_{k}\right)$$

After the model training, we evaluated the performance of the proposed scheme with various performance parameters with the testing data. The metrics are described in the following. During the model testing, true positives are when the items in the dataset are positive and they are predicted to be positive; and true negatives are when the items in the dataset are negative and they are predicted to be negative. A false negative is positive but is predicted as negative; and a false positive is negative but is predicted as positive. ALL consists of the combination of all above parameters. Further, in the testing process, the test dataset was used, and parameters such as recall, accuracy, precision, and F1_score were calculated.

They are measured as follows [18]:

(9) $$\begin{array}{c} \mathrm{Precision}(P)=\frac{\mathrm{tp}}{\mathrm{tp}+\mathrm{fp}},\;\mathrm{Recall}(R)=\frac{\mathrm{tp}}{\mathrm{tp}+\mathrm{fn}} \end{array}$$

(10) $$\begin{array}{c} \mathrm{Accuracy}(A)=\frac{\mathrm{tp}+\mathrm{tn}}{\mathrm{ALL}},\;F1\_\mathrm{score}=\frac{2\times P\times R}{P+R} \end{array}$$

We achieved accuracy for GANB, RFA, and KNN of 92.2%, 89.9%, and 78.5%, respectively. Using Equations (9) and (10) for the proposed stacked model, the precision, recall, accuracy, and F1_score were 0.981, 0.984, 0.925, and 0.983, respectively.

## 4. The Proposed Approach

The section presents the proposed model and the working process of the proposed model in terms of the algorithm.

### 4.1. Dataset Description

To train the stacked model PSD detection approach, we have used the PPMI dataset [17]. The dataset consists of 3 files of patients’ medical history and characteristics and 19 files of the patients’ imaging biomarker data, medical history, and motor and non-motor assessments. Imaging biomarker files consist of the values computed from the findings in the medical imaging techniques, and these values are stored in comma-separated files. The medical imaging techniques included dopamine transporter scan (DaTSCAN), diffusion tensor imaging (DTI), MRI, and PET. For DaTSCAN, the data have the DATSCAN_LIGAND, DATSCAN_CAUDATE_R, DATSCAN_CAUDATE_L, DATSCAN_PUTAMEN_R, DATSCAN_PUTAMEN_L, DATSCAN_PUTAMEN_R_ANT, and DATSCAN_PUTAMEN_L_ANT features available for the patients. These features correspond to the portion of caudate putamen, a central component of basal ganglia that can be used to observe motor, cognition, and speech functions. Using these features, we can know about the disease symptoms, and by observing the differences in these biomarkers, we can help identify the disease’s severity. Similar data are available for each of the imaging techniques. Motor and non-motor assessments were also included to increase the robustness of the model.

These files contain data about each visit of the patient over a period of time. A steady decline was observed in the visits in comparison to the first baseline visit by all patients. All the data were combined into a single file, and only those patient visits with sufficient data available were considered. In this study, we considered 100 patients data’ from each hospital visit. These data were analyzed to maintain the minimum threshold for the model to generalize. A patient number was assigned to each patient to keep track of his or her identity. The merged data contain 1596 columns, including the column for patient identity. A detailed description of the number of patients involved in each visit is listed in Table 2.

### 4.2. Data Preprocessing and Proposed Stacked Model

We propose an ML-based model to classify PSD patients and HC subjects from the dataset. ML techniques can be used for medical assistance with identifying diseases. We used the PPMI dataset, which contains biomarker-defined cohorts, to analyze and study PSD progression using imaging biomarkers. It has biological parameters that can be quantified using different modalities or their combinations, such as clinical, imaging, genetic, and biospecimen PSD markers. The objective of measuring medical signs is to measure the effects of treatment for a patient or measure the presence and progress of a disease. PPMI used biomarkers to establish biomarker cohorts and find longitudinal progression biomarkers to support future PSD identification and treatments [19].

The approach discussed in the model is summarized below.

- Three individual machine learning models were used to classify the patients based on the biomarkers available using the PPMI dataset in the form of comma-separated-values files.
- Then, a meta learner was created to combine the results of each individual learner, and we used that to predict the stacked model.
- Finally, we evaluated the performance of each of the models on both training and testing sets.

**Figure 2:**
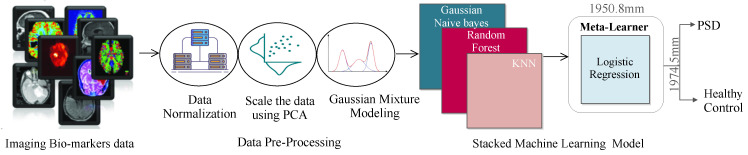
The proposed approach.

We propose a stacked ML approach to differentiate HC patients from PSD patients. Stacking allows solving a problem using different ML models by combining the different learning abilities of models. The stacked model uses several classification models and uses their output labels as input for the meta classifier, as shown in Figure 2. A meta learner improves the quality of the results by combining usually weak models and having relatively low complexity. It combines the predictions made by each weak model and uses its prediction probability as input for the meta learner to compute the overall prediction of the stacked model.

**Figure 3:**
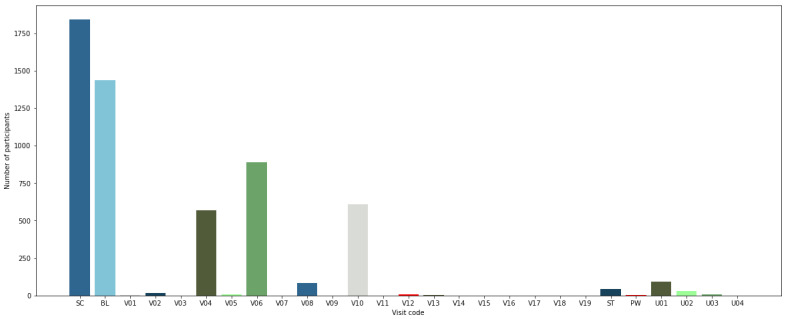
Visit distribution of participants in the study.

Figure 3 shows the visits and the number of participants in each study. Biomarkers over several visits were measured to identify the progression of PSD and aid the treatment and identification. We have included the biomarkers from the cohorts that had data from several visits to identify and learn about the progression of the disease over time. There was a decline in the number of participants from the baseline visit. The dataset has progression biomarkers from several categories. We included imaging biomarkers for our study and merged all of them as input data for each visit. We used data from these visits and then constructed the input data by converting them into a single time series dataset of all visits to observe and analyze the progression of PSD at subsequent visits at the hospital gradually.

After preparing the time series data, we fed those data into the stacked model. The ML models can learn from them and are not susceptible to noise, missing values, or unusable formats. We transformed the data using min-max normalization to scale the data. The entire dataset was scaled into a smaller range so that multiple attributes on different scales would not dilute the model’s accuracy. We fetched the maximum and minimum values for each biomarker attribute and scaled them down to the new range. The training and testing data were split using the 80–20 ratio, and then only the training data were scaled using the min-max scaler. The scaling factor obtained from the training data was then applied to test data while making inferences and predictions. After normalization, we applied dimensionality reduction techniques such as FA, PCA, and GMM. FA uses agglomerative clustering, which is an unsupervised clustering technique to group together features that are similar and recursively merges pair of clusters of features to reduce the number of features. Similarly, we applied PCA to reduce the dimensions of the dataset by converting the large set of attributes into a smaller one, while keeping important information about the dataset. The PCA is responsible for the trade-off between the accuracy and complexity of the ML model. In the first step, it calculated a covariance matrix to identify the relationships between the different attributes in the dataset. Some attributes have high correlations that contain redundant data.

Then, eigenvectors and eigenvalues were computed from the matrix to find the principal components. These principal components are combinations of highly uncorrelated attributes and can compress most information into a few components. It reduced the size of the dataset without losing the dependencies. Geometrically, principal components signify the direction of data with maximum variance. After forming the feature vectors, we recast them along with the principal components. The GMM probabilistic method was used to cluster the input data. The assumptions involved for GMM are that input data were gathered from a mixture of Gaussian distributions. Due to the assumption that the data belong to a Gaussian distribution, the reduced data from GMM do not completely represent the original data.

**Figure 4:**
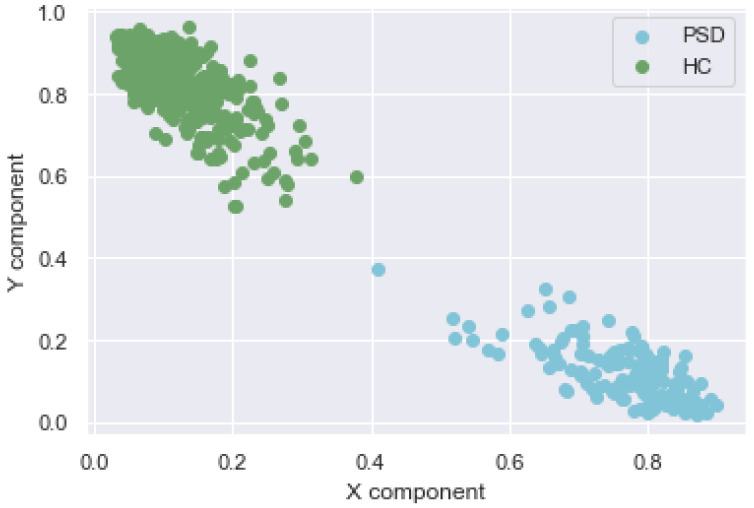
Feature space for disease progression.

Among the discussed dimensionality reduction techniques, we used the FA technique, which gives the minimum reconstruction error, and thus it gives the best representation of the original data. Figure 4 shows the features obtained after clustering using feature agglomerative clustering, and we can see a clear distinction between the attributes of PSD and HC participants. The progression of the disease was measured using the UPDRS scale to estimate the disease progression of the PSD patient. By using the six segments involved in the UPDRS scale against the response to physical treatment and medication, we could predict the extent of severity of the disease by analyzing the scores of the patient in each visit. Therefore, we could analyze the changes in scale and monitor the reaction to the treatment provided to the patient. We can observe a distinct progression in the feature space from the healthy patient in the bottom right to increased disease progression as we move towards the left and top in the projected feature space. Similar feature expansion could be carried out to understand the varying degrees of the disease based on the UPDRS score. Inside the cluster of PSD patients, the points on the right side represent the patients having mild symptoms and at the beginning of the disease. As we move towards left and upwards withing the PSD cluster, the disease severity increases. By plotting the features of a newer patient onto this feature space, one can estimate whether the patient has PSD or HC, and furthermore, if the patient has the disease, the severity can be estimated. To observe a proper transition between the different UPDRS scores requires more data, which would reveal a distinct transition from initial disease to a severe case.

After the data preprocessing, we analyzed the correlations between the biomarkers in the progression of PSD. We proposed a supervised ML algorithm to classify and identify PSD patients using biomarkers. We experimented with several combinations of the base model and meta learners to classify the PSD patients correctly. Based on that, we proposed a combination for the stacked model that outperformed other combinations, achieving 92.5% accuracy. We propose a stacked model with GANB, RFA classifier, and KNN classifier as base learners, and logistic regression as the meta learner to train the model.

### 4.3. Gaussian Naive Bayes

The first base model in our stacked model is GANB. It is a statistical classifier that performs probabilistic prediction. It predicts class membership probabilities. GANB assumes that each of the classes follows a Gaussian distribution and extracts the independent features. To define the distribution for each class we find the mean and standard deviation for each class and use them to fit the model. The F1_score and accuracy for the GANB model were 0.982 and 92.2%, respectively.

### 4.4. Random Forest Classifier

The next base model in the proposed stacked model is the RFA classifier. It is a supervised learning algorithm which is an ensemble of decision trees. The algorithm is based on the concept that the accuracy of the ML model improves with the combination of learning models. The F1_score and accuracy for the RFA model were 0.958 and 89.9%, respectively.

### 4.5. K-Nearest Neighbor Classifier

The last base model in the proposed stacked model is the KNN classifier. It is also a supervised learning algorithm that uses the similarity between existing classes and includes the new class into the most similar category to the available class category. We use uniform weights: each neighbor is weighted equally. KNN is a nonparametric classification algorithm, which means that the model does not make any underlying assumptions about the distribution, in contrast to other algorithms, such as Gaussian mixture models, which work on the assumption of a Gaussian distribution of the data. The output for the KNN algorithm is a class membership. It considers the plurality of the votes of its neighbors, and the object is assigned to the class which is most common among its K neighbors. The F1_score and accuracy for the KNN model were 0.795 and 78.5%, respectively.

### 4.6. Proposed Stacked Model

Based on all the base models and their output classifications, we took them as input and trained our stacked model. We used the outputs of GANB, RFA classifier, and KNN to compute the final prediction. We used logistic regression as a meta learner to produce the final predictions. It uses stratified K-folds cross-validation, a variant of the K-fold cross-validation technique that gives stratified folds. Each of these folds is done by keeping aside a percentage of samples for each class. The stacked model outperformed each of the base models. The F1_score and accuracy for the proposed stacked model were 0.983 and 92.5%, respectively. The implementation of each of the ML models was done using Python and sklearn libraries.

### 4.7. Training Algorithm for Stacked Model

Algorithm 1 shows the steps for training a stacked ensemble model to classify the patients in HC and PSD. We used base-level classifiers individually to train the model, and then took the output from each classifier as an input for the meta learner classifier. In our model, we use logistic regression, and each class is given equally important weightage. We then took classification output from the proposed stacked model to get the final output.
**Algorithm 1** Meta learning from base-level classifiers and the final ensemble classifier for prediction**Input:** Training data DA = {a_i_, b_i_}${}_{k=1}^{N}$**Output:** Ensemble classifier E1:**procedure**Learn from base level classifiers(:)2:    **for** tr = 1 to TR **do**3:        Learn from E_tr_ based on DA4:    **end for**5:**end procedure**6:**procedure**Build new dataset for predictions(:)7:    **for** *k* = 1 to n **do**8:        DA_e_ = {ai${}^{\prime }$, b_i_}, where ai${}^{\prime }$= {e_1_(a_i_), ……,e_TR_(a_i_)}9:    **end for**10:**end procedure**11:**procedure**Learn from Meta-Classifiers(:)12:    Learn E based on D_e_13:    Return E14:**end procedure**

### 4.8. Proposed Algorithm

The execution process of the trained model is presented in Algorithm 2. Initially, the data collected using PPMI are used as the input [17]—the imaging biomarkers are the input; then it extracts the features and preprocess the data. We applied preprocessing step on the input data to avoid influences of any outliers or noise in the dataset. After that, we applied FA to reduce the dataset’s dimensionality and identify whether we could establish a relationship between the PSD and HC classes. It helped to improve the interpretability and accuracy of the models. Afterwards, we trained the data with three types of algorithm: KNN, RFA, and GANB. Then, we fed our data into model training for the stacked model, where the model was trained by using the meta learner as logistic regression. The significant outcomes are presented using precision, recall, F1_score, and accuracy.
**Algorithm 2** Execution process of the proposed model**Input:** Imaging biomarkers dataset is collected from PPMI**Output:** Classification of PSD and HC subject1:**procedure**Pre-Processing data(:)2:    X $\overset{}{\leftarrow }$ Input data3:    X = MIN-MAX Normalization (X)4:    X = FA (X)5:**end procedure**6:**procedure**Model Training phase(:)7:    GANB $\overset{}{\leftarrow }$ GANB (X)8:    RFA $\overset{}{\leftarrow }$ RFA algorithm (X)9:    KNN $\overset{}{\leftarrow }$ K Neighbors algorithm (X)10:    Classifier algorithm $\overset{}{\leftarrow }$ [GANB, RFA, KNN]11:    Meta Learning classifier algorithm $\overset{}{\leftarrow }$ Logistic regression algorithm ()12:    Stacked $\overset{}{\leftarrow }$ Stacking classifiers(Classifier, Meta-Learning classifier)13:**end procedure**14:**procedure**Testing and evaluation Phase(:)15:    **for** <i in models> **do**16:        Model. fit (X)17:        Predicted Output $\overset{}{\leftarrow }$ Model.predict()18:        Accuracy $\overset{}{\leftarrow }$ Model.evaluate()19:    **end for**20:**end procedure**

## 5. Performance Evaluation

In this paper, we proposed a stacked ML algorithm to classify HC and PSD patients. The stacked model uses predictions from different ML models on the same dataset. Each base model makes its predictions, and then the prediction probability is used as an input for the meta learner to combine the prediction capabilities. Stacking models are useful when the errors in predictions made by different models are uncorrelated or have a low correlation. The stacked model can learn from the dataset and perform classification by combining the inferences from multiple models that could not have been possible by individual base models.

**Figure 5:**
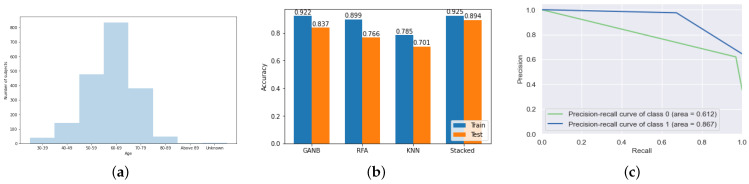
(**a**) Age distribution of participants in the study. (**b**) Comparative analysis of different classifiers. (**c**) Cumulative gain for the stacked ML model.

Figure 5a shows the distribution of the participants in terms of age parameters. The PPMI dataset contains information of patients with PSD, HC, prodroma, and scans without evidence of dopaminergic deficit (SWEDD). Among the given data, we based our study on the data which consisted of 154 HC participants and 294 PSD participants. Sex-wise, 33% of the participants were women, and 67% of the participants were men. The ages of the participants represented are as of 2022. PSD generally onsets around the age of 60 years and above. The young-onset of PSD (YOPSD) is when it occurs in people younger than 50 years of age. For YOPSD, a combination of genetics and environmental reasons is believed to be at fault [20].

Table 3 shows the accuracy and F1_score for each of the three folds used for the K-fold cross-validation for the training data. The final training accuracy and F1_score report the averages of results obtained in each fold. Table 4 shows the accuracy of each model, along with the standard deviation. We trained the multiple base models and meta learners with different combinations. In our proposed model, we used the combination of GANB, RFA classifier, and KNNs. We then passed the prediction probability from each of these models as input to the meta learner to compute the output classification for a stacked model. We used logistic regression as the meta learner for our model. The stacked model outperformed each of the base models, achieving an accuracy of 92.5%. The GANB, RFA classifier, and KNNs had accuracies of 92.2%, 89.9%, and 78.5% respectively, as shown in Figure 5b.

The test accuracies obtained for the GANB, RFA classifier, and KNN models were 83.7%, 76.6%, and 70.1%, respectively. We support the use of the stacked model over each of the individual models because of the improved performance of the stacked model on the testing data. The stacked model outperformed every other model, achieving a test accuracy of 89.4%. Even though the stacked model performed similarly to the GANB classifier for the validation data, the stacked model demonstrated more generalization capabilities and better performance than every other model. The stacked model based its decisions on learning from each of the individual models; therefore, it improved the overall performance. We also calculated the binomial proportion confidence interval for the classification accuracy of the test data, and we obtained accuracy in the range 87.7–90.9% with a 95% confidence level for the assumed Gaussian distribution. The F1_score for the given model was 98.3%, and the specificity for the given model was 88.2%.

The F1_score, calculated using the harmonic mean of precision and recall, represents the measure of the model’s accuracy. The GANB, RFA classifier, and KNN had F1_scores of 0.982, 0.958, and 0.795, respectively. The stacked model had an F1_score of 0.983.

**Figure 6:**
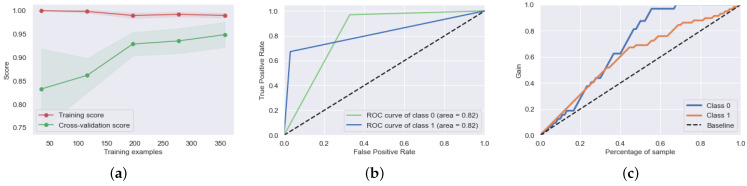
(**a**) Learning curve for the stacked ML model made to classify HC and PSD patients. (**b**) Receiver operating characteristic curve for the stacked ML model. (**c**) Precision–recall curve for the stacked ML model.

Figure 6a shows the learning curve for the stacked model. It reflects the performance of the stacked model on the training and testing data as the number of training instances changes. We infer that the model’s performance improves with the increase in number of training instances. Three-fold cross validation was used to calculate the average score over all the training subsets. The stacked model improved the overall accuracy compared to the base model. It also showed better generalization when predicting and learning PSD patient markers. The stacked model started to overfit once over 350 iterations were used.

Figure 6b shows the receiver operating characteristic curve (ROC) of the proposed stacked model. It was used to measure the performance of classification at various threshold levels against the baseline level. Here, class 0 refers to HC and class 1 represents the PSD class. The area under the ROC curve (AUC) refers to the measure of the classification ability of a model. Higher AUC values depict better performance of the classification model in distinguishing between the classes. The AUC for both the classes in the proposed stacked model was 82%. It represents the trade-off for a classification model between sensitivity and specificity. The proposed model outperformed the others in learning and classifying the HC patients and can be used as a differential aid when classifying patients with few of the physical symptoms, alongside clinical tests.

Figure 6c shows the precision–recall curve for the proposed stacked classifier. It was used to measure the trade-off between the precision and recall values for varying threshold levels. High values of area under the precision–recall curve represent low false-positive rates and low false-negative rates, which relate to accurate results by the classifier. The areas of the precision–recall curve for class 0 (HC) and class 1 (PSD) are 61.2% and 86.7%. The proposed stacked model resulted in a lower area under the curve for the HC patient class as compared to the PSD class. Thus, the proposed model showed a better precision–recall trade-off in classifying correct labels for PSD as compared to HC patients. The precision–recall curve helps with identifying a class imbalance due to the lack of quality data, which may decrease the accuracy of a model.

Figure 5c shows the cumulative gain curve for the proposed stacked model. The cumulative gain curve is an evaluation measure that shows the percentage of overall number of cases in a category gained by targeting a percentage of the total cases. Cumulative gain in a specified decile represents the ratio of cumulative number of outputs up to that decile to the total number of outputs. This can be used to decide what population we should sample to get the desired sensitivity for the proposed stacked model. We could choose an appropriate value from the cumulative gains to employ this for our proposed stacked model on an extended dataset.

While observing the results and checking the performance of the model in terms of wrongly predicted classifications, it was found that the data for the subjects had either missing or inconsistent values for some of the entries. The remaining portion of the wrong predictions can be attributed to the capability of the model to generalize. The performance of the model is expected to improve if provided more data.

## 6. Conclusions

PSD is a progressive neurodegenerative disease of the brain, where the dopamine level can damage the nerve cells inside the central brain. In this paper, we measured the disease progression by the loss of dopamine level inside the brain. Knowing the disease progression helps to identify the risk levels of the patients to take preventive measures early. We designed an algorithm for preprocessing the data to avoid noise and missing values, and scale data into a smaller range. Afterwards, we described the execution process of the proposed model using an algorithm. The proposed model was built using several ML algorithms and combined their results in a meta learning process. This stacked model gives significantly superior results as compared to the existing state-of-the-art work: the model achieved 92.5% accuracy when aiming to correctly diagnose patients’ diseases.

In the future, we will train our model using deep learning techniques with imaging biomarkers that could improve its performance. Further, we will integrate blockchain technology for exchanging Parkinson’s data securely and reliably among all healthcare centers for overseas diagnosis too. 

## Figures and Tables

**Table 1:** Comparative analysis of the existing imaging biomarker-based schemes and the proposed scheme.

Author	Year	Objective	Algorithm	Result	Pros	Cons
Nunes et al. [12]	2019	To discriminate PSD, HC and Alzheimer’s disease data using retina texture biomarkers	SVM	Classification of PSD, HC, and Alzheimer’s disease accuracy = 82.9%	Classify PSD, AD and HC from the data	Lower Accuracy
Pereira et al. [13]	2019	To classify the PSD patient using medical imaging	AI algorithms	Classification accuracy of PSD and HC subject accuracy = 65.7%	Classify PD, and HC subject from SPECT and MRI images	Lower accuracy
Mangesius et al. [14]	2020	Proposed decision algorithm to classify parkinsonism with imaging biomarkers	Decision tree algorithm	Classification of parkinsonism accuracy = 83.7%	Classify PD, MSA and PSP from MRI imaging biomarker	Does not given the classification of HC subject
Lin et al. [15]	2020	To detect the disease progression of PSD patient	Biomedical method to extract the data	-	Provide a disease progression of 3-year data of PSD patient	Does not given a classification of HC subject
Kathuria et al. [16]	2021	To classify and diagnosis of PSD patient from atypical parkinsonism and HC using MRI and F-DOPA PET imaging	Biomedical method to extract the features from imaging modality	MRI_VenoBOLD Accuracy: 95% Sensitivity: 88.4% Specificity: 66.7%, MRI_SWI Sensitivity: 93% Specificity: 80%	Diagnosis of idiopathic PSD and atypical parkinsonism with nigrosome imaging	Small sample size of atypical parkinsonism subject.
The proposed scheme	2021	Classification of PSD and HC patients using imaging biomarkers data	Stacked ML model	Accuracy = 92.5%, F1_score = 98%, Precision = 98% and Recall = 97%	Diagnosis of PSD and HC using imaging biomarkers, Measures the disease progression of PSD patient	-

**Table 2:** Dataset description about the number of participants involved in each visit.

Visit Code	Visit Description	Number of Subjects	Visit Code	Visit Description	Number of Subjects
SC	Screening	1844	V12	Month 60	6
BL	Baseline	1435	V13	Month 72	4
V01	Month 3	4	V14	Month 84	0
V02	Month 6	19	V15	Month 96	0
V03	Month 9	0	V16	Month 108	0
V04	Month 12	570	V17	Month 120	0
V05	Month 18	6	V18	Month 132	0
V06	Month 24	891	V19	Month 144	0
V07	Month 30	0	ST	Symptomatic Therapy	44
V08	Month 36	84	PW	Premature Withdrawal	2
V09	Month 42	0	U01	Unscheduled Visit 01	94
V10	Month 48	608	U02	Unscheduled Visit 02	29
V11	Month 54	0	U03	Unscheduled Visit 03	8

**Table 3:** Accuracy and F1_score for K fold cross validation.

Fold	Accuracy				F1_Score			
	**GANB**	**RFA**	**KNN**	**Stacked**	**GANB**	**RFA**	**KNN**	**Stacked**
1st Fold	91.1	90.3	75.1	91.0	0.977	0.947	0.781	0.980
2nd Fold	92.3	89.5	79.2	94.3	0.983	0.960	0.803	0.984
3rd Fold	92.6	89.9	79.7	92.2	0.984	0.967	0.801	0.985
Average	92.2	89.9	78.5	92.5	0.982	0.958	0.795	0.983

**Table 4:** Accuracies of various ML models.

Model	Accuracy	Deviation
GANB	92.2%	+/−2.6%
RFA classifier	89.9%	+/−6.6%
KNN	78.5%	+/−2.9%
Proposed stacked model	92.5%	+/−2.0%

## Data Availability

No data are associated with this research work.
